# Alterations of Cerebral Blood Flow and Its Connectivity in Olfactory-Related Brain Regions of Type 2 Diabetes Mellitus Patients

**DOI:** 10.3389/fnins.2022.904468

**Published:** 2022-07-11

**Authors:** Wei Luo, Jie Wang, Mimi Chen, Shanlei Zhou, Datong Deng, Fujun Liu, Yongqiang Yu

**Affiliations:** ^1^Department of Imaging, Chaohu Hospital of Anhui Medical University, Hefei, China; ^2^Department of Radiology, The First Affiliated Hospital of Anhui Medical University, Hefei, China; ^3^Research Center of Clinical Medical Imaging, Hefei, China; ^4^Department of Endocrinology, The First Affiliated Hospital of Anhui Medical University, Hefei, China

**Keywords:** arterial spin labeling, type 2 diabetes mellitus, cerebral blood flow, olfactory, cognitive, connectivity

## Abstract

To investigate the alteration of cerebral blood flow (CBF) and its connectivity patterns in olfactory-related regions of type 2 diabetes mellitus (T2DM) patients using arterial spin labeling (ASL). Sixty-nine patients with T2DM and 63 healthy controls (HCs) underwent ASL scanning using 3.0T magnetic resonance imaging. We compared the CBF values of the olfactory-related brain regions between the two groups and analyzed the correlation between their changes and clinical variables. We also used these regions as seeds to explore the differences in CBF connectivity patterns in olfactory-related brain regions between the T2DM patients and HCs. Compared with the HC group, the CBF of the right orbital part of the inferior frontal gyrus (OIFG), right insula, and bilateral olfactory cortex was decreased in the T2DM patients. Moreover, the duration of the patients was negatively correlated with the CBF changes in the right OIFG, right insula, and right olfactory cortex. The CBF changes in the right OIFG were positively correlated with the Self-Rating Depression Scale scores, those in the right insula were negatively correlated with the max blood glucose of continuous glucose, and those in the right olfactory cortex were negatively correlated with the mean blood glucose of continuous glucose. In addition, the T2DM patients also showed decreased CBF connectivity between the right OIFG and the left temporal pole of the middle temporal gyrus and increased CBF connectivity between the right medial orbital part of the superior frontal gyrus and the right orbital part of the superior frontal gyrus and between the right olfactory cortex and the bilateral caudate and the left putamen. Patients with T2DM have decreased CBF and altered CBF connectivity in multiple olfactory-related brain regions. These changes may help explain why olfactory dysfunction occurs in patients with T2DM, thus providing insights into the neuropathological mechanism of olfactory dysfunction and cognitive decline in T2DM patients.

## Introduction

Diabetes mellitus is one of the fastest-growing diseases worldwide, both in relative and absolute numbers. The International Diabetes Federation^1^ reported a total of 537 million people with diabetes worldwide in 2021, and within 10 years, this number is expected to reach 600 million, with type 2 diabetes mellitus (T2DM) representing the major type; thus, T2DM will pose a huge social, economic and public health burden. As a systemic disease, T2DM can lead to chronic damage and dysfunction of the nervous system. Several studies have shown that T2DM accelerates age-related cognitive decline and patients with T2DM are more likely to develop mild cognitive impairment and Alzheimer’s disease (McCrimmon et al., 2012; Koekkoek et al., 2015; Biessels et al., 2020). Accurate diagnosis and characterization of clinically relevant cognitive impairment in diabetes is important in clinical consultation because a bidirectional relationship occurs between diabetes management and cognition. Poor blood glucose control increases the risk of cognitive dysfunction, and conversely, cognitive dysfunction can adversely affect diabetes management. Identifying individuals who may develop dementia in the early stages of the disease is critical to the prognosis of patients (American Diabetes Association, 2019; LeRoith et al., 2019).

The endocrine and olfactory systems are also closely related, and studies have reported deficits in multiple olfactory domains in patients with T2DM, such as elevated odor detection thresholds (Lietzau et al., 2018), decreased odor identification (Sanke et al., 2014), and increased risk of anosmia (Faour et al., 2022). Moreover, previous studies have indicated that olfactory dysfunction in T2DM is closely related to cognitive impairment and early changes in the olfactory system may be induced by T2DM before cognitive decline (Zhang et al., 2018; Lietzau et al., 2020). Therefore, olfactory deficits in T2DM patients may be an early marker of cognitive decline (Zhang et al., 2018). Unfortunately, dysfunction of the olfactory system is usually not accurately detected in time compared to dysfunction of other sensory systems, and only a minority of patients with olfactory impairment are aware of such impairment before testing (Kondo et al., 2020). Therefore, an objective assessment of olfactory function is essential, especially for those at risk, to accurately describe the nature and severity of olfactory hyposmia (Doty, 2017).

Many studies have begun to focus on olfactory-related brain changes in T2DM patients using neuroimaging methods. These studies have shown that patients with T2DM are often accompanied by atrophy in olfactory-related brain regions, such as the orbitofrontal cortex (OFC) and hippocampus, and evidence has shown that atrophy in these regions is the earliest neuroanatomical change in Alzheimer’s disease (Brundel et al., 2014; Yoon et al., 2017; Rosenberg et al., 2019; Sanjari Moghaddam et al., 2019). Moreover, a task-state study showed that before structural brain abnormalities were observed, T2DM patients showed reduced odor-induced brain activation and abnormal functional connectivity in olfactory-related brain regions during olfactory tasks (Zhang et al., 2018). However, previous resting-state functional magnetic resonance connectivity studies have been performed almost exclusively by measuring blood oxygen level-dependent (BOLD) signals and have rarely investigated abnormal changes in cerebral blood flow (CBF) in olfactory-related brain regions and its connectivity patterns in T2DM patients. While CBF is a single physiological parameter, CBF may be more closely related to brain metabolism (Zou et al., 2009). The distribution pattern of CBF is not random but rather is related to differences in the anatomical distribution of brain vessels or the innervation pattern of brain vessels. Neuronal activity is the main energy expenditure process of the brain, and regional CBF will be adjusted according to the level of energy expenditure. The activity of neuronal circuits is the main factor determining CBF changes (Melie-Garcia et al., 2013). Moreover, most of the energy expenditure of the brain is attributed not to external stimuli but internal activity in the resting state (Raichle and Mintun, 2006). Therefore, studying synchronous CBF fluctuations in olfactory-related brain regions can be used to measure functional connectivity in these brain regions at rest.

Based on the above findings, this study explored the changes in CBF and its connectivity patterns in olfactory-related brain regions in T2DM patients using arterial spin labeling (ASL) techniques. In addition, a correlation analysis between the CBF changes in these regions and clinical variables in T2DM patients was also performed to provide a new method for the clinical prediction and evaluation of cognitive decline in T2DM. We hypothesized that the functions and functional connectivity of olfactory-related brain regions are abnormal in T2DM and characterized by changes in CBF and its connectivity pattern in olfactory-related brain regions in patients.

## Materials and Methods

### Participants

In this study, T2DM patients were recruited from inpatients and outpatients of the Department of Endocrinology of the First Affiliated Hospital of Anhui Medical University. Inclusion criteria included an age range of 25–65 years, at least 6 years of education, and right-handedness. The diagnosis of T2DM followed the WHO diagnostic criteria for T2DM (Barzilay et al., 1999). Healthy controls (HCs) were recruited from community advertisements and matched with T2DM patients for age, gender, and years of education. For all included subjects, the exclusion criteria included the following: (1) dementia and psychiatric disorders; (2) thyroid dysfunction; (3) severe cerebrovascular disease; (4) history of traumatic brain injury and surgery; (5) pregnancy or any magnetic resonance imaging (MRI) contraindications; and (6) history of nasal abnormalities (acute phase of infection, history of nasal surgery). HCs with fasting plasma glucose (FPG) ≥ 7.0 mmol/L and a history of abnormal blood glucose were excluded. The study was approved by the Research Ethics Committee of the First Affiliated Hospital of Anhui Medical University, and all participants voluntarily signed informed consent forms.

A total of 104 patients in the T2DM group and 89 volunteers in the HC group were recruited for this study. Thirty-five patients in the T2DM group and 26 volunteers in the HC group were excluded due to one or more factors, including poor image quality (20 T2DM patients and 16 volunteers in the HC group), failure to complete all cognitive tests and olfactory tests (8 T2DM patients and 7 volunteers in the HC group), cerebrovascular disease (1 volunteer in the HC group), history of traumatic brain injury (1 volunteer in the HC group) and claustrophobia (1 volunteer in the HC group), failure to complete all MRI sequence scans (6 T2DM patients), and history of nasal surgery (1 T2DM patient). Thus, the final data analysis included 69 T2DM patients (mean age ± SD, 46.4 ± 8.2 years; 27.5% female) and 63 volunteers in the HC group (mean age ± SD, 45.1 ± 7.7 years; 31.7% female).

### Data Collection

Demographic data and biochemical measurements were collected from subjects in both groups. Fundus examination results were collected from all T2DM patients to diagnose the presence of diabetic retinopathy (DR). In addition, continuous glucose data were also collected from T2DM patients who wore continuous glucose monitoring (CGM) devices (29 individuals). CGM wear data collected for at least 7 days were considered acceptable and included mean blood glucose (gluMean), max blood glucose (gluMax), minimum blood glucose (gluMin), coefficient of variation (CV), and average amplitude of glucose excursion (MAGE).

### Cognitive Testing and Olfactory Function Assessment

We administered comprehensive neuropsychological tests to all subjects. The Mini-Mental State Examination (MMSE) and the Montreal Cognitive Assessment (MoCA) were used to assess the general cognitive functioning of all subjects. Participants were evaluated on multiple cognitive subdomains, including verbal memory, executive function, word fluency, working memory, information processing speed, and motor response speed, using the following tests: Auditory Verbal Learning Test (AVLT), Trail Making Test-A (TMT-A), Verbal Fluency Test (VFT), Digit Span Test (DST), and Symbol Digital Modalities Test (SDMT). The Self-Rating Depression Scale (SDS) and Self-Rating Anxiety Scale (SAS) were used to evaluate the degree of depression and anxiety and reflect their psychological status. The olfactory function evaluation tool was the China Smell Identification Test (CSIT), which was designed by the Chinese Academy of Sciences to suit the Chinese population (Feng et al., 2019). All participants completed all tests in a fixed order within 2 h.

### Magnetic Resonance Imaging Acquisition

Brain MRI scans were performed using a 3.0T MR scanner (Discovery MR750w, General Electric, Milwaukee, WI, United States) with a 24-channel head coil. The high-resolution structural images of the brain were acquired using a brain volume (BRAVO) sequence with the following parameters: sagittal slices = 188, slice thickness = 1 mm, echo time (TE) = 3.2 ms, repetition time (TR) = 8.5 ms, inversion time (TI) = 450 ms, flip angle = 12°, field of view (FOV) = 256 mm × 256 mm, matrix = 256 × 256, and acquisition time = 296 s. CBF maps were acquired by the ASL sequence with 3D fast spin-echo acquisition and background suppression. The parameters of the sequence are as follows: axial slices = 50, slice thickness = 3 mm, TR = 5070 ms, TE = 11.5 ms, Post labeling delay time = 2025 ms, FOV = 240 mm × 240 mm, matrix = 128 × 128, sample points = 512, Number of excitation = 3, and scan duration = 294 s. During the scanning period, the subjects were required to relax, close their eyes, remain awake, and fix their head with a foam pad to avoid head movement.

### Image Preprocessing

Individual ASL difference images were calculated from control images of each participant after subtracting the corresponding label images, and subsequently, the CBF maps of each participant were derived from the ASL difference images (Xu et al., 2010). The CBF maps were processed further with Statistical Parametric Mapping (SPM) software (version 12)^2^. The CBF maps were normalized to Montreal Neurological Institute (MNI) template space using high-resolution individual structure images as intermediates (Yezhuvath et al., 2012). Non-brain tissue was then removed, and normalized CBF values were obtained by dividing the CBF value of each voxel by the global mean CBF value, which is a more sensitive metric than absolute CBF because it decreases data noise induced by intersubject variations in global CBF (Aslan and Lu, 2010). Finally, all normalized CBF images were spatially smoothed using an isotropic Gaussian kernel of 8 mm × 8 mm × 8 mm full-width at half maximum (FWHM) to improve the signal-to-noise ratio of CBF maps.

### Normalized Cerebral Blood Flow Comparisons in Olfactory-Related Brain Regions

Based on previous neuroimaging studies (Felix et al., 2021; Chen et al., 2022), a total of 18 olfactory-related regions of interest (ROIs, 9 left and 9 right) were selected for this study, including the amygdala, OFC regions (including the orbital parts of the superior, middle and inferior frontal gyri; the medial orbital part of the superior frontal gyrus, MOSFG; the olfactory cortex), hippocampus, parahippocampal gyrus, and insula. These ROIs were then defined using the AAL template in the WFU PickAtlas software^3^. Finally, the normalized CBF values of ROIs for all subjects were extracted using Data Processing and Analysis for (Resting-State) Brain Imaging (DPABI)^4^ software for comparisons between groups. Independent samples *t*-tests were used for comparative analyses in SPSS 22.0 software and the false discovery rate (FDR) method was used to correct for multiple comparisons, with *P* < 0.05 considered statistically significant. In addition, to further explore the effects of vascular lesions and treatment modalities on CBF in olfactory-related brain regions of T2DM, we divided patients in the T2DM group into subgroups with or without DR and subgroups with different treatments. Fourteen of the patients in the T2DM group had DR, and the remaining patients did not have DR. Twenty-nine patients with T2DM were treated with non-drug therapy, 21 patients required antidiabetic drug therapy, and the remaining 19 patients were treated with insulin. Then, compared the CBF of olfactory-related brain regions was compared between the subgroups.

### Correlation Between the Cerebral Blood Flow in Olfactory-Related Brain Regions and Clinical Parameters in Patients With T2DM

For the olfactory-related brain regions with significant differences in CBF between groups, we also performed partial correlation analyses between the CBF changes in these brain regions and clinical variables in T2DM patients. These clinical variables included disease duration, biochemical examinations, and neuropsychological tests, with age, years of education, and sex as covariates. *P* < 0.05 indicated statistically significant differences.

### Cerebral Blood Flow Connectivity in Olfactory-Related Brain Regions

Cerebral blood flow connectivity is a covariant feature between brain regions by calculating the correlation coefficient of CBF in different brain regions of a group of subjects. In this study, 18 olfactory-related brain regions were used as seeds to explore the CBF connectivity patterns of olfactory-related brain regions of T2DM patients and HCs. Specifically, we used SPM12 to construct multiple linear regression models in the two groups of subjects to calculate the partial correlation coefficient (CBF connectivity) between the CBF in each seed region and CBF in all other voxels of the brain in the two groups of subjects, with age, years of education and sex as covariates. Multiple comparisons were corrected using a family wise error (FWE) method (*P* < 0.05). For each ROI, the CBF connectivity maps of the T2DM and HC groups were merged into a single spatial mask to correlate the CBF of each voxel in that spatial mask with the CBF of the ROI in either of the two groups. For any pair of voxels, the difference in CBF connectivity between the two groups is indicated by the fact that the CBF correlation of this pair of voxels may have different slopes in the two groups. To analyze the changes in CBF connectivity in olfactory-related brain regions in T2DM patients, we compared the CBF at the voxel level between the two groups using two-sample *t*-tests within the spatial mask of the merged CBF connectivity map after controlling for age, years of education, and sex (Zhu et al., 2015). The FWE method was used for multiple comparisons (*P* < 0.05, cluster size threshold was set as 120).

### Statistical Analysis

Demographic data, biochemical examinations, and neuropsychological test data were statistically analyzed using SPSS 22.0 software. The Kolmogorov–Smirnov method was used to test the normality of the data. The normal distribution of data was expressed as mean ± standard deviation. The data with non-normal distribution were represented by median (25th and 75th percentile). Normally distributed measurement data were compared using two-sample *t*-tests. Non-parametric test (Mann–Whitney *U* test) was used for comparison between groups of non-normal distribution data. Categorical variables (for example, sex) were compared between groups using chi-square tests. Two-tailed *P* < 0.05 was set as the significance level.

## Results

### Demographic and Neuropsychological Measurement Data

A total of sixty-nine patients with T2DM and sixty-three volunteers in the HC group were included in this study. Demographic and biochemical examination data are shown in Table 1. BMI and FPG values were higher in the T2DM patients than in the HC subjects (*P* < 0.05). Statistically significant differences were not observed in blood pressure or smoking and alcohol habits between the two groups (*P* > 0.05). The results of the neuropsychological tests are shown in Table 2. The CSIT results showed that the olfactory identification function decreased in the T2DM group compared with the HC group (*P* < 0.05). The MoCA, DST, SDMT, and VFT results were lower in the T2DM group than in the HC group, while the TMT-A, SDS, and SAS results were higher in the T2DM group (*P* < 0.05). The results of the cognitive function test showed that the MMSE and AVLT values did not differ significantly between the two groups (*P* > 0.05).

**TABLE 1:** Characteristics of the T2DM patients and healthy control (HC).

Index	T2DM	HCs	*P*-value
**Demographic factors**			
*N* (Female/Male)^#^	19/50	20/43	0.700
Age (years)	46.4 ± 8.2	45.1 ± 7.7	0.350
Education (years)	12.7 ± 3.5	13.6 ± 4.0	0.150
BMI (kg/m^2^)	25.3 ± 3.4	24.0 ± 2.5	0.012*
Alcohol consumption (*n*, %)^#^	19, 27.5%	12,19.0%	0.181
Smoking habits (*n*, %)^#^	22, 31.9%	11, 17.5%	0.056
Systolic blood pressure (mmHg)	128.6 ± 15.0	124.6 ± 14.8	0.126
Diastolic blood pressure (mmHg)	83.2 ± 9.7	80.6 ± 10.5	0.132
MoCA < 26 (*n*, %)^#^	29, 42.0%	15, 23.8%	0.027*
**Diabetes-related characteristics**			
FPG (mmol/L)	9.7 ± 3.1	5.1 ± 0.4	<0.001***
Treatment (non, ant, and ins)	29, 21, 19		
DR (*n*, %)	14, 20.3%		
Duration of diabetes (months)	36 (12, 108)		
HbA1c (%)	9.5 ± 2.2		
**Continuous glucose monitoring**			
gluMean (mmol/L)	8.6 ± 1.6		
gluMax (mmol/L)	19.2 ± 3.6		
gluMin (mmol/L)	3.5 ± 0.9		
CV	0.36 ± 0.07		
MAGE (mmol/L)	6.1 ± 1.3		

T2DM, type 2 diabetes mellitus; HCs, healthy controls; BMI, body mass index; FPG, fasting plasma glucose; treatment: non, non-drug therapy; ant, antidiabetic drug therapy; ins, insulin therapy; DR, diabetic retinopathy; HbA1c, glycated hemoglobin; continuous glucose: gluMean, mean blood glucose; gluMax, max blood glucose; gluMin, minimum blood glucose; CV, coefficient of variation; MAGE, mean amplitude of glycemic excursions. ^#^Pearson χ^2^ analysis for dichotomous variables, *P-value < 0.05, ***P-value < 0.001.

**TABLE 2:** Olfactory test and cognitive assessment in patients with T2DM and HC.

Index	T2DM	HCs	*P*-value
CSIT	31.3 ± 4.6	33.0 ± 3.8	0.024*
MMSE	28.7 ± 1.8	29.0 ± 1.4	0.222
MoCA	25.5 ± 3.6	26.8 ± 2.6	0.019*
AVLT-immediate	9.0 ± 1.7	9.1 ± 2.1	0.767
AVLT-delayed	9.2 ± 3.6	9.8 ± 3.0	0.267
AVLT-recognition	13.9 ± 1.3	13.9 ± 1.8	0.850
TMT-A (s)	39.2 ± 13.4	9.1 ± 2.1	0.018*
DST-forward	8.0 ± 1.6	8.4 ± 1.5	0.100
DST-backward	5.3 ± 1.6	6.0 ± 1.8	0.012*
SDMT	47.5 ± 11.8	53.5 ± 13.2	0.007**
VFT-animal	17.8 ± 4.7	20.4 ± 5.0	0.002**
VFT-fruit	13.1 ± 3.0	14.4 ± 3.5	0.033*
SDS	6.2 ± 3.5	4.5 ± 3.8	0.009**
SAS	34.5 ± 6.5	32.3 ± 5.3	0.039*

T2DM, type 2 diabetes mellitus; HCs, healthy controls; CSIT, Chinese Smell Identification Test; MMSE, Mini-Mental State Examination; MoCA, Montreal Cognitive Assessment; AVLT-immediate, Auditory Verbal Learning Test Immediate Memory; AVLT-delayed, Auditory Verbal Learning Test Delayed Memory; AVLT-recognition, Auditory Verbal Learning Test Oral Recognition; TMT-A, Trail Making Test-A; DST-forward, Digit Span Test-forward; DST-backward, Digit Span Test-backward; SDMT, Symbol Digital Modalities Test; VFT-animal, Verbal Fluency Test-animal; VFT-fruit, Verbal Fluency Test-fruit; SDS, Self-Rating Depression Scale; SAS, Self-Rating Anxiety Scale. *P-value < 0.05, **P-value < 0.01.

### Group Differences in Normalized Cerebral Blood Flow

**FIGURE 1:**
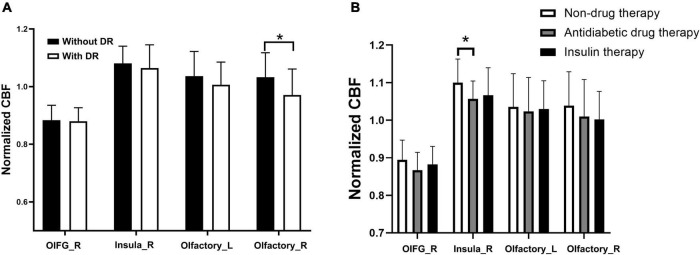
Stratified analysis of CBF in olfactoryrelated brain regions in T2DM subgroups. **(A)** Subgroups with or without DR. **(B)** Subgroups with different treatments. *P-value < 0.05. OIFG_R, right orbital part of the inferior frontal gyrus; Insula_R, right insula; Olfactory_L, left olfactory cortex; olfactory_R, right olfactory cortex; without DR, without diabetic retinopathy; DR, diabetic retinopathy; normalized CBF, normalized cerebral blood flow.

A comparative analysis revealed that among the 18 olfactory-related brain regions, the CBF in multiple olfactory-related brain regions presented significant differences between the two groups (Table 3). Specifically, the CBF in the right orbital part of the inferior frontal gyrus (OIFG), right insula, and bilateral olfactory cortex was significantly decreased in patients with T2DM compared with the HC group (*P* < 0.05, FDR corrected). The stratified analysis revealed that T2DM patients with DR had lower CBF in the four differential brain regions than those without DR, and the difference in CBF in the right olfactory cortex was statistically significant (Figure 1A, *P* < 0.05). The results of the analysis for the different treatment modalities revealed that T2DM patients requiring antidiabetic drug therapy and insulin therapy had lower CBF in the four differential brain regions than non-drug therapy patients and T2DM patients requiring antidiabetic drug therapy had significantly lower CBF in the right insula than non-drug therapy patients (Figure 1B, *P* < 0.05).

**TABLE 3:** Comparison of cerebral blood flow (CBF) in olfactory-related brain areas between the T2DM and HC.

Regions		T2DM (*SD*)	HCs (*SD*)	*T*	Original *P-*values	FDR-corrected *P*-values
Amygdala	Left	0.852(0.064)	0.854(0.058)	–0.210	0.834	0.834
	Right	0.837(0.070)	0.858(0.063)	–1.683	0.095	0.342
OIFG	Left	0.904(0.052)	0.914(0.066)	–1.070	0.286	0.644
	Right	0.883(0.051)	0.917(0.059)	–3.542	0.001	0.009*
MOSFG	Left	1.108(0.068)	1.119(0.090)	–0.785	0.434	0.654
	Right	1.107(0.067)	1.115(0.073)	–0.717	0.475	0.654
OMFG	Left	0.900(0.069)	0.886(0.074)	1.100	0.273	0.644
	Right	0.908(0.071)	0.918(0.061)	–0.877	0.382	0.654
OSFG	Left	0.936(0.061)	0.934(0.084)	–0.500	0.618	0.654
	Right	0.941(0.064)	0.944(0.084)	–1.244	0.216	0.644
Hippocampus	Left	0.904(0.064)	0.913(0.055)	–0.887	0.377	0.654
	Right	0.915(0.066)	0.923(0.071)	–0.703	0.483	0.654
Insula	Left	1.013(0.066)	1.019(0.059)	–0.510	0.611	0.654
	Right	1.077(0.064)	1.107(0.067)	–2.625	0.010	0.045*
Olfactory cortex	Left	1.030(0.085)	1.068(0.082)	–2.609	0.010	0.045*
	Right	1.020(0.089)	1.076(0.085)	–3.706	< 0.001	0.005*
Parahippocampal	Left	0.899(0.054)	0.894(0.042)	0.518	0.605	0.654
	Right	0.926(0.057)	0.932(0.059)	–0.617	0.538	0.654

CBF, cerebral blood flow; T2DM, type 2 diabetes mellitus; HCs, healthy controls; SD, standard deviation; OIFG, orbital part of the inferior frontal gyrus; MOSFG, medial orbital part of the superior frontal gyrus; OMFG, orbital part of the middle frontal gyrus; OSFG, orbital part of the superior frontal gyrus. *P-value < 0.05 (FDR corrected).

### Correlation Between Cerebral Blood Flow Changes and Clinical Variables and Neuropsychological Tests

**FIGURE 2:**
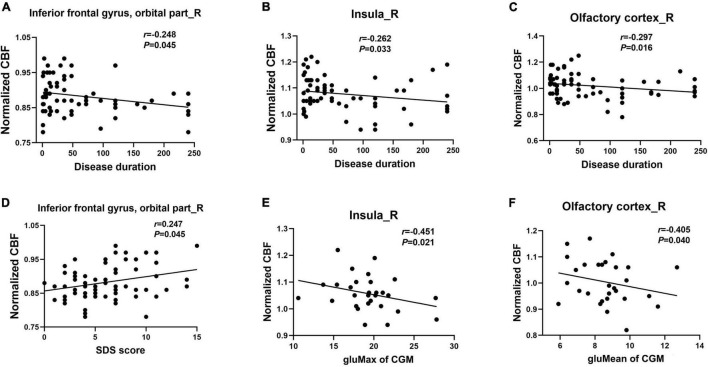
Correlation of the CBF changes in olfactory brain regions with clinical parameters and neuropsychological tests in patients with T2DM **(A–F)**. SDS, Self-Rating Depression Scale; gluMax, max blood glucose; gluMean, mean blood glucose.

A correlation analysis showed that after controlling for age, sex, and years of education, the CBF changes in olfactory-related brain regions in patients with T2DM were correlated with clinical variables and neuropsychological test results (Figure 2 and Supplementary Table 1, *P* < 0.05). Specifically, the disease duration of T2DM patients was negatively correlated with the CBF changes in the right OIFG (*r* = −0.248, *P* = 0.045), right insula (*r* = −0.262, *P* = 0.033), and right olfactory cortex (*r* = −0.297, *P* = 0.016). CBF changes in the right OIFG were positively correlated with the SDS score (*r* = 0.247, *P* = 0.045); CBF changes in the right insula were negatively correlated with the gluMax of CGM (*r* = −0.451, *P* = 0.021); and CBF changes in the right olfactory cortex were negatively correlated with gluMean of CGM in patients with T2DM (*r* = −0.405, *P* = 0.040). However, significant correlations were not observed between the CBF changes in these olfactory-related brain regions and the CSIT scores.

### Cerebral Blood Flow Connectivity Patterns in the T2DM Patients and Healthy Control Group

**FIGURE 3:**
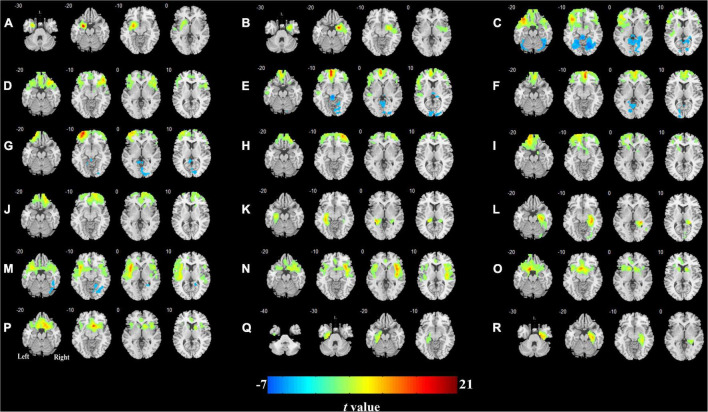
Cerebral blood flow (CBF) connectivity patterns in olfactory-related brain regions in HCs. **(A)** Left amygdala; **(B)** right amygdala; **(C)** left orbital part of the inferior frontal gyrus; **(D)** right orbital part of the inferior frontal gyrus; **(E)** left medial orbital part of the superior frontal gyrus; **(F)** right medial orbital part of the superior frontal gyrus; **(G)** left orbital part of the middle frontal gyrus; **(H)** right orbital part of the middle frontal gyrus; **(I)** left orbital part of the superior frontal gyrus; **(J)** right orbital part of the superior frontal gyrus; **(K)** left hippocampus; **(L)** right hippocampus; **(M)** left insula; **(N)** right insula; **(O)** left olfactory cortex; **(P)** right olfactory cortex; **(Q)** left parahippocampal gyrus; **(R)** right parahippocampal gyrus.

**FIGURE 4:**
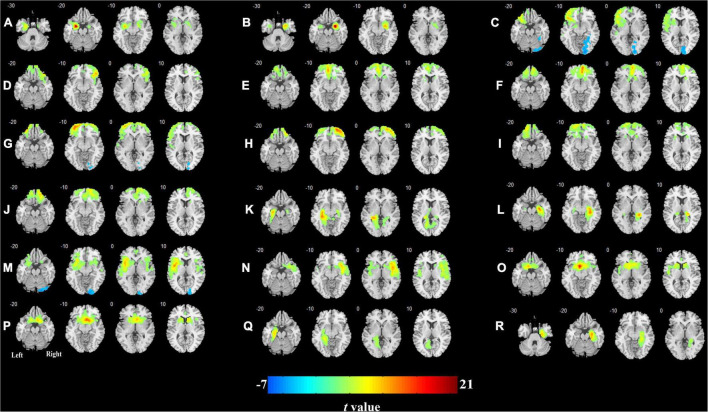
Cerebral blood flow connectivity patterns in olfactory-related brain regions in T2DM. **(A)** Left amygdala; **(B)** right amygdala; **(C)** left orbital part of the inferior frontal gyrus; **(D)** right orbital part of the inferior frontal gyrus; **(E)** left medial orbital part of the superior frontal gyrus; **(F)** right medial orbital part of the superior frontal gyrus; **(G)** left orbital part of the middle frontal gyrus; **(H)** right orbital part of the middle frontal gyrus; **(I)** left orbital part of the superior frontal gyrus; **(J)** right orbital part of the superior frontal gyrus; **(K)** left hippocampus; **(L)** right hippocampus; **(M)** left insula; **(N)** right insula; **(O)** left olfactory cortex; **(P)** right olfactory cortex; **(Q)** left parahippocampal gyrus; **(R)** right parahippocampal gyrus.

The CBF connectivity maps of the 18 ROIs for the two groups are shown in Figures 3, 4 (*P* < 0.05, FWE corrected). When the right orbital part of the middle frontal gyrus (OMFG, Figures 3H, 4H), the left orbital part of the superior frontal gyrus (OSFG, Figures 3I, 4I), the right OSFG (Figures 3J, 4J), the left olfactory cortex (Figures 3O, 4O), and the right parahippocampal gyrus (Figures 3R, 4R) were used as seeds, the CBF connectivity patterns were approximately equivalent between the two groups, whereas when the remaining ROIs were used as seeds, the CBF connectivity patterns were significantly different between the two groups.

### Differences in Cerebral Blood Flow Connectivity in Olfactory-Related Brain Regions Between the Two Groups

**FIGURE 5:**
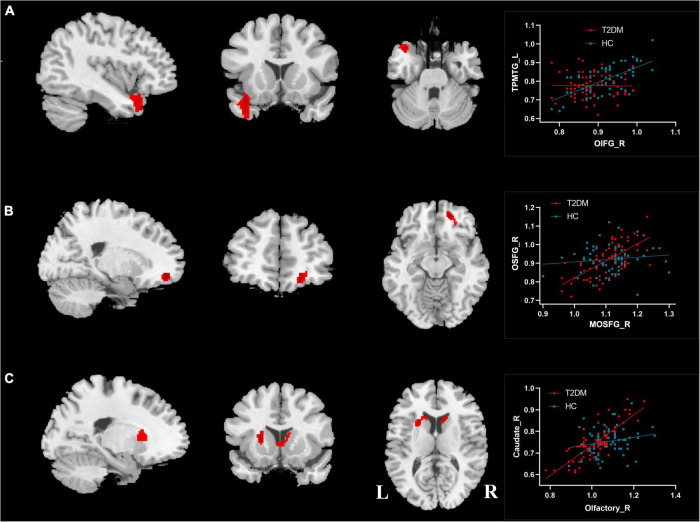
Differences of CBF connectivity in olfactory brain regions between T2DM patients and HC group. **(A)** Right orbital part of the inferior frontal gyrus; **(B)** right medial orbital part of the superior frontal gyrus; **(C)** right olfactory cortex. OIFG_R, right orbital part of the inferior frontal gyrus; TPMTG_L, left temporal pole of middle temporal gyrus; MOSFG_R, right medial orbital part of the superior frontal gyrus; OSFG_R, right orbital part of the superior frontal gyrus; L, left; R, right.

Among the 18 ROIs, significant differences in CBF connectivity were observed among the three brain regions of the right OIFG, right MOSFG, and right olfactory cortex (*P* < 0.05, FWE corrected, Table 4 and Figure 5). Compared with the HC group (*r* = 0.61, *P* < 0.001; *r* = 0.16, *P* = 0.221), the T2DM group (*r* = −0.11, *P* = 0.381; *r* = 0.72, *P* < 0.001) showed significantly decreased and increased positive CBF connectivity between the right OIFG and left temporal pole of the middle temporal gyrus (TPMTG, Figure 5A) and between the right MOSFG and right OSFG (Figure 5B), respectively. When the right olfactory cortex was used as the seed point, the positive CBF connectivity between this brain region and the bilateral caudate and the left putamen significantly increased in the T2DM group (*r* = 0.80, *P* < 0.001) compared with the HC group (*r* = 0.25, *P* = 0.059) (Figure 5C).

**TABLE 4:** Differences in CBF connectivity in olfactory brain regions between patients with T2DM and HCs.

No.	Seed regions	Differential brain regions	Cluster size	*t-*value of the peak point	MNI coordinates of the peak point (*x*-, *y*-, *z*-)
(1)	Right OIFG	Left TPMTG	265	4.3	−38, 20, −34
(2)	Right MOSFG	Right OSFG	120	4.6	20, 48, −12
(3)	Right olfactory cortex	Bilateral caudate, left putamen	355	4.2	−22, 14, 8

*CBF, cerebral blood flow; T2DM, type 2 diabetes mellitus; HCs, healthy controls; MNI, Montreal Neurological Institute; OIFG, orbital part of the inferior frontal gyrus; TPMTG, temporal pole of the middle temporal gyrus; MOSFG, medial orbital part of the superior frontal gyrus; OSFG, orbital part of the superior frontal gyrus.*

## Discussion

In this study, we demonstrated that multiple cognitive domains and olfactory identification function were impaired in T2DM patients. More importantly, ASL was used to explore the changes in CBF and CBF connectivity in olfactory-related brain regions in patients with T2DM. We found decreased CBF in the right OIFG, right insula, and bilateral olfactory cortex in patients with T2DM. A stratified analysis showed that patients with DR had lower CBF in these brain regions than those without DR, and CBF in these brain regions was lower in T2DM patients requiring antidiabetic drug therapy and insulin therapy than in non-drug therapy patients. In patients with T2DM, these CBF changes in olfactory-related brain regions were significantly associated with disease duration, CGM, and SDS scores. In particular, this study is the first to use ASL-MRI to establish the olfactory CBF connectivity pattern in T2DM patients. We found abnormal CBF connectivity in the right OIFG, right MOSFG, and right olfactory cortex in patients with T2DM.

In the present study, we administered a comprehensive neuropsychological test to all subjects to assess multiple cognitive domains of the subjects. We found that T2DM patients had poorer executive function, working memory, information processing speed, motor response speed, language function, and olfactory identification function than HCs, which is consistent with the findings of many previous studies (Callisaya et al., 2019; Moran et al., 2019). The decrease in olfactory identification in T2DM patients may be caused by neurodegenerative diseases in the olfactory-related brain regions including the primary olfactory cortex (piriform cortex, entorhinal cortex, and amygdala) and neocortex (OFC). These olfactory-related brain regions are also important for higher brain functions such as cognition, memory, and emotion and are susceptible to neuropathological abnormalities (Felix et al., 2021; Chen et al., 2022).

Previous neuroimaging studies have relied heavily on volumetric measurements of diseased brain regions, which reflect advanced changes in neurodegeneration (Segura et al., 2013; Kjelvik et al., 2014; Dintica et al., 2019). This study explored the resting-state CBF of olfactory-related brain regions in T2DM patients with ASL technology and found that the CBF of the ROIFG, right insula, and bilateral olfactory cortex was reduced in T2DM patients. The brain relies on the continuous supply of glucose as its main source of energy and metabolism, and changes in blood glucose concentrations directly affect brain function. Previous studies have shown that acute or chronic hyperglycemia is accompanied by reduced CBF (Shemesh et al., 2019). The regional cerebral cortex is hypoperfused in T2DM patients, and chronic hyperglycemia disrupts the blood–brain barrier, leading to permanent brain cell damage, which demonstrates that altered cerebral hemodynamics is a potential mechanism of cognitive decline in T2DM patients (Jansen et al., 2016). The CBF of several olfactory-related brain regions decreased in T2DM patients in this study, and the decreased CBF in these brain regions may cause abnormal brain function, which leads to hyposmia in T2DM patients. DR is a neurovascular complication of diabetes, and microangiopathy of the retina may be an indirect marker of microcirculatory changes in the brain. Notably, T2DM patients with DR showed significantly worse odor identification function than T2DM patients without DR (Sanke et al., 2014). This result is similar to our finding that patients with DR have more severe functional abnormalities in olfactory-related brain regions. We also found that T2DM patients requiring antidiabetic drugs and insulin therapy had lower CBF in olfactory-related brain regions. Further, we found that T2DM patients requiring antidiabetic drugs had a longer duration of disease than Non-drug patients (Supplementary Table 2, *P* < 0.001). This may be related to the fact that these patients with advanced diabetes tend to have more severe brain abnormalities due to their continued deterioration of islet function (Reinke et al., 2022).

Further correlation analysis revealed that the CBF changes in the right OIFG, right insula, and right olfactory cortex of T2DM patients were all negatively correlated with the duration of diabetes. Thus, the longer the duration of the disease, the lower the blood perfusion of these olfactory brain regions in patients. Scholars Moheet et al. (2015) and Cheng et al. (2021) also found that the duration of diabetes plays an important role in the development of brain dysfunction, and the longer the duration of the disease, the greater the risk of impaired brain function in patients. We also found that CBF changes in the right OIFG of T2DM patients were positively correlated with depression scores. The right OIFG is a core component of the default mode network and is associated with a variety of brain functions, including emotional control and self-consciousness. Abnormal spontaneous neural activity in the right OIFG may contribute to impaired emotion regulation in patients with major depressive disorder with somatic symptoms (Yan et al., 2019). The prevalence of depression in patients with T2DM is quite high, which may be related to cerebral microvascular dysfunction in patients with T2DM (Zhou et al., 2021). This result is similar to the finding presented here that abnormal blood perfusion in the right OIFG may contribute to mood disorders in patients with T2DM.

In this study, the CBF changes in the right insula of T2DM patients were negatively correlated with the gluMax of CGM, and the CBF changes in the right olfactory cortex were negatively correlated with gluMean of CGM. Compared to chronic hyperglycemia, acute hyperglycemia may have a greater impact on patients, with acute blood glucose fluctuations leading to more severe persistent activation of chronic inflammation, endothelial cell DNA damage, and oxidative stress (Wang et al., 2021). CGM can provide clinicians with a rich and accurate basis for adjusting treatment strategies to better control diabetes and delay the occurrence and development of diabetic complications. The results of this study also demonstrate the importance of glucose monitoring and glucose control to protect the normal brain function of the olfactory brain region in T2DM patients. However, it must be noted that only a portion of T2DM patients in this study had satisfactory CGM data, which may reduce the statistical validity of certain findings. Future studies must further validate the reliability of this result with multicenter and large samples.

However, the present study did not find that changes in CBF in olfactory brain regions of diabetic patients correlated with olfactory tests and cognitive tests. Many studies have reported reduced CBF in patients with T2DM, although the results have been mixed across studies (Xia et al., 2015; Cui et al., 2017; Bangen et al., 2018). And only a few studies have reported reduced CBF in olfactory-related brain regions (hippocampus) in patients with T2DM, which found a strong association between reduced CBF and poorer memory in non-demented older adults with T2DM (Bangen et al., 2018). Another study reported that patients with T2DM had increased right insula perfusion, improved cognitive ability, and improved to a greater extent than non-diabetic controls after insulin administration, and that these insulin-induced changes were associated with vasodilatation in the middle cerebral artery territory (Novak et al., 2014). These studies suggest that inadequate perfusion may underlie the cognitive decline in patients with T2DM. Therefore, more indicators of olfactory function (such as olfactory threshold and olfactory discrimination function) need to be measured in future studies with longitudinal follow-up of large samples to better investigate how reduced CBF in olfactory-related brain regions of T2DM patients affects olfactory function in T2DM and its relationship with cognitive impairment.

The CBF connectivity was measured by calculating the partial correlation coefficient of CBF between pairs of brain regions in the study. Few studies have investigated the functional connectivity of olfactory-related brain regions in patients with T2DM. Functional magnetic resonance studies have found that functional connectivity between the olfactory-related brain region and the right insula is impaired in obese T2DM patients before cognitive decline (Zhang et al., 2019). In this study, we found that CBF connectivity between the right OIFG and the left TPMTG was decreased in T2DM patients. The OIFG and MTG are important brain regions for emotion control (Jastorff et al., 2016; Yan et al., 2019), suggesting that reduced CBF connectivity between these two brain regions may be associated with abnormal emotions in patients with T2DM. We also observed increased CBF connectivity between the right MOSFG and the right OSFG in patients with T2DM and between the right olfactory cortex and the striatum (bilateral caudate, left putamen). The MOSFG and the OSFG are part of the prefrontal lobe, which is an important part of the default network (Zald et al., 2014). The olfactory cortex is the primary olfactory center, which acts to perceive odors and is also involved in odor discrimination and odor-induced emotional changes (Pashkovski et al., 2020). The increased CBF connectivity in these brain regions may be a compensatory mechanism exhibited by T2DM patients to maintain normal cognitive and olfactory functions. In summary, the changes in CBF connectivity in multiple olfactory-related brain regions in T2DM patients may play a role in the reduced olfactory function, cognitive dysfunction, and emotional abnormalities observed in T2DM patients.

Some limitations of our study should be noted. First, the present study did not find a significant correlation between CBF changes in these olfactory-related brain regions and the results of olfactory tests. Second, the reliability of measuring functional connectivity between brain regions may be affected by the relatively low signal-to-noise ratio of current ASL images; thus, the signal-to-noise ratio of ASL images should be optimized in future studies.

## Conclusion

We found reduced CBF and altered CBF connectivity in multiple olfactory-related brain regions in T2DM patients. These changes may be the cause of olfactory dysfunction in T2DM patients and could represent a potential new neuroimaging marker for predicting and evaluating olfactory dysfunction in T2DM; moreover, they may play a role in elucidating the neuropathological mechanisms of cognitive function, olfactory dysfunction, and emotional abnormalities in T2DM patients.

## Data Availability Statement

The original contributions presented in this study are included in the article/Supplementary Material, further inquiries can be directed to the corresponding author.

## Ethics Statement

The studies involving human participants were reviewed and approved by the Research Ethics Committee of the First Affiliated Hospital of Anhui Medical University. The patients/participants provided their written informed consent to participate in this study.

## Author Contributions

WL, JW, MC, and YY designed the study. WL, JW, and MC conducted the study and analyzed the data. WL, JW, MC, SZ, DD, and FL were responsible for data collection. WL wrote the manuscript. YY was responsible for obtaining funding for the study and editing drafts of the manuscript. All authors contributed to and approved the final manuscript.

## Conflict of Interest

The authors declare that the research was conducted in the absence of any commercial or financial relationships that could be construed as a potential conflict of interest.

## Publisher’s Note

All claims expressed in this article are solely those of the authors and do not necessarily represent those of their affiliated organizations, or those of the publisher, the editors and the reviewers. Any product that may be evaluated in this article, or claim that may be made by its manufacturer, is not guaranteed or endorsed by the publisher.

## Footnotes

1
https://diabetesatlas.org/

2
http://www.fil.ion.ucl.ac.uk/spm

3
https://www.nitrc.org/projects/wfu_pickatlas

4
http://rfmri.org/dpabi

## References

[B1] American Diabetes Association (2019). 12. Older Adults: standards of medical care in diabetes-2019. *Diabetes Care* 42 (Suppl. 1), S139–S147. 10.2337/dc19-S012 30559238

[B2] Aslan S. Lu H. (2010). On the sensitivity of ASL MRI in detecting regional differences in cerebral blood flow. *Magn. Reson. Imaging* 28 928–935. 10.1016/j.mri.2010.03.037 20423754 PMC2912434

[B3] Bangen K. J. Werhane M. L. Weigand A. J. Edmonds E. C. Delano-Wood L. Thomas K. R. (2018). Reduced regional cerebral blood flow relates to poorer cognition in older adults with type 2 Diabetes. *Front. Aging Neurosci.* 10:270. 10.3389/fnagi.2018.00270 30250430 PMC6139361

[B4] Barzilay J. I. Spiekerman C. F. Wahl P. W. Kuller L. H. Cushman M. Furberg C. D. (1999). Cardiovascular disease in older adults with glucose disorders: comparison of American Diabetes Association criteria for diabetes mellitus with WHO criteria. *Lancet* 354 622–625. 10.1016/s0140-6736(98)12030-5 10466662

[B5] Biessels G. J. Nobili F. Teunissen C. E. Simo R. Scheltens P. (2020). Understanding multifactorial brain changes in type 2 diabetes: a biomarker perspective. *Lancet Neurol.* 19 699–710. 10.1016/S1474-4422(20)30139-3 32445622

[B6] Brundel M. Kappelle L. J. Biessels G. J. (2014). Brain imaging in type 2 diabetes. *Eur. Neuropsychopharmacol.* 24 1967–1981. 10.1016/j.euroneuro.2014.01.023 24726582

[B7] Callisaya M. L. Beare R. Moran C. Phan T. Wang W. Srikanth V. K. (2019). Type 2 diabetes mellitus, brain atrophy and cognitive decline in older people: a longitudinal study. *Diabetologia* 62 448–458. 10.1007/s00125-018-4778-9 30547230

[B8] Chen B. Espin M. Haussmann R. Matthes C. Donix M. Hummel T. (2022). The effect of olfactory training on olfaction, cognition, and brain function in patients with mild cognitive impairment. *J. Alzheimers Dis.* 85 745–754. 10.3233/JAD-215257 34864678

[B9] Cheng P. Song S. Li Y. Zhang Y. Yi J. Xu X. (2021). Aberrant functional connectivity of the posterior cingulate cortex in type 2 diabetes without cognitive impairment and microvascular complications. *Front. Endocrinol.* 12:722861. 10.3389/fendo.2021.722861 34759889 PMC8573207

[B10] Cui Y. Liang X. Gu H. Hu Y. Zhao Z. Yang X. Y. (2017). Cerebral perfusion alterations in type 2 diabetes and its relation to insulin resistance and cognitive dysfunction. *Brain Imaging Behav.* 11 1248–1257. 10.1007/s11682-016-9583-9 27714551 PMC5653700

[B11] Dintica C. S. Marseglia A. Rizzuto D. Wang R. Seubert J. Arfanakis K. (2019). Impaired olfaction is associated with cognitive decline and neurodegeneration in the brain. *Neurology* 92 e700–e709. 10.1212/WNL.0000000000006919 30651382 PMC6382360

[B12] Doty R. L. (2017). Olfactory dysfunction in neurodegenerative diseases: Is there a common pathological substrate? *Lancet Neurol.* 16 478–488. 10.1016/S1474-4422(17)30123-0 28504111

[B13] Faour M. Magnan C. Gurden H. Martin C. (2022). Olfaction in the context of obesity and diabetes: insights from animal models to humans. *Neuropharmacology* 206:108923. 10.1016/j.neuropharm.2021.108923 34919903

[B14] Felix C. Chahine L. M. Hengenius J. Chen H. Rosso A. L. Zhu X. (2021). Diffusion tensor imaging of the olfactory system in older adults with and without hyposmia. *Front. Aging Neurosci.* 13:648598. 10.3389/fnagi.2021.648598 34744681 PMC8569942

[B15] Feng G. Zhuang Y. Yao F. Ye Y. Wan Q. Zhou W. (2019). Development of the Chinese smell identification test. *Chem. Senses* 44 189–195. 10.1093/chemse/bjz006 30715263

[B16] Jansen J. F. van Bussel F. C. van de Haar H. J. van Osch M. J. Hofman P. A. van Boxtel M. P. (2016). Cerebral blood flow, blood supply, and cognition in Type 2 Diabetes Mellitus. *Sci. Rep.* 6:160003. 10.1038/s41598-016-0003-6 27920431 PMC8276879

[B17] Jastorff J. De Winter F. L. Van den Stock J. Vandenberghe R. Giese M. A. Vandenbulcke M. (2016). Functional dissociation between anterior temporal lobe and inferior frontal gyrus in the processing of dynamic body expressions: insights from behavioral variant frontotemporal dementia. *Hum. Brain. Mapp.* 37 4472–4486. 10.1002/hbm.23322 27510944 PMC6867423

[B18] Kjelvik G. Saltvedt I. White L. R. Stenumgard P. Sletvold O. Engedal K. (2014). The brain structural and cognitive basis of odor identification deficits in mild cognitive impairment and Alzheimer’s disease. *BMC Neurol.* 14:168. 10.1186/s12883-014-0168-1 25154749 PMC4236673

[B19] Koekkoek P. S. Kappelle L. J. van den Berg E. Rutten G. E. Biessels G. J. (2015). Cognitive function in patients with diabetes mellitus: guidance for daily care. *Lancet Neurol.* 14 329–340. 10.1016/S1474-4422(14)70249-2 25728442

[B20] Kondo K. Kikuta S. Ueha R. Suzukawa K. Yamasoba T. (2020). Age-related olfactory dysfunction: epidemiology, pathophysiology, and clinical management. *Front. Aging Neurosci.* 12:208. 10.3389/fnagi.2020.00208 32733233 PMC7358644

[B21] LeRoith D. Biessels G. J. Braithwaite S. S. Casanueva F. F. Draznin B. Halter J. B. (2019). Treatment of diabetes in older adults: an endocrine society* Clinical Practice Guideline. *J. Clin. Endocrinol. Metab.* 104 1520–1574. 10.1210/jc.2019-00198 30903688 PMC7271968

[B22] Lietzau G. Davidsson W. Ostenson C. G. Chiazza F. Nathanson D. Pintana H. (2018). Type 2 diabetes impairs odour detection, olfactory memory and olfactory neuroplasticity; effects partly reversed by the DPP-4 inhibitor Linagliptin. *Acta Neuropathol. Commun.* 6:14. 10.1186/s40478-018-0517-1 29471869 PMC5824492

[B23] Lietzau G. Nystrom T. Wang Z. Darsalia V. Patrone C. (2020). Western diet accelerates the impairment of odor-related learning and olfactory memory in the mouse. *ACS Chem. Neurosci.* 11 3590–3602. 10.1021/acschemneuro.0c00466 33054173 PMC7645872

[B24] McCrimmon R. J. Ryan C. M. Frier B. M. (2012). Diabetes and cognitive dysfunction. *Lancet* 379 2291–2299. 10.1016/S0140-6736(12)60360-2 22683129

[B25] Melie-Garcia L. Sanabria-Diaz G. Sanchez-Catasus C. (2013). Studying the topological organization of the cerebral blood flow fluctuations in resting state. *Neuroimage* 64 173–184. 10.1016/j.neuroimage.2012.08.082 22975159

[B26] Moheet A. Mangia S. Seaquist E. R. (2015). Impact of diabetes on cognitive function and brain structure. *Ann. N. Y. Acad. Sci.* 1353 60–71. 10.1111/nyas.12807 26132277 PMC4837888

[B27] Moran C. Beare R. Wang W. Callisaya M. Srikanth V. Alzheimer’s Disease Neuroimaging I. (2019). Type 2 diabetes mellitus, brain atrophy, and cognitive decline. *Neurology* 92 e823–e830. 10.1212/WNL.0000000000006955 30674592 PMC7987953

[B28] Novak V. Milberg W. Hao Y. Munshi M. Novak P. Galica A. (2014). Enhancement of vasoreactivity and cognition by intranasal insulin in type 2 diabetes. *Diabetes Care* 37 751–759. 10.2337/dc13-1672 24101698 PMC3931384

[B29] Pashkovski S. L. Iurilli G. Brann D. Chicharro D. Drummey K. Franks K. M. (2020). Structure and flexibility in cortical representations of odour space. *Nature* 583 253–258. 10.1038/s41586-020-2451-1 32612230 PMC7450987

[B30] Raichle M. E. Mintun M. A. (2006). Brain work and brain imaging. *Annu. Rev. Neurosci.* 29 449–476. 10.1146/annurev.neuro.29.051605.112819 16776593

[B31] Reinke C. Buchmann N. Fink A. Tegeler C. Demuth I. Doblhammer G. (2022). Diabetes duration and the risk of dementia: a cohort study based on German health claims data. *Age Ageing* 51:afab231. 10.1093/ageing/afab231 34923587 PMC8753043

[B32] Rosenberg J. Lechea N. Pentang G. N. Shah N. J. (2019). What magnetic resonance imaging reveals - A systematic review of the relationship between type II diabetes and associated brain distortions of structure and cognitive functioning. *Front. Neuroendocrinol.* 52:79–112. 10.1016/j.yfrne.2018.10.001 30392901

[B33] Sanjari Moghaddam H. Ghazi Sherbaf F. Aarabi M. H. (2019). Brain microstructural abnormalities in type 2 diabetes mellitus: a systematic review of diffusion tensor imaging studies. *Front. Neuroendocrinol.* 55:100782. 10.1016/j.yfrne.2019.100782 31401292

[B34] Sanke H. Mita T. Yoshii H. Yokota A. Yamashiro K. Ingaki N. (2014). Relationship between olfactory dysfunction and cognitive impairment in elderly patients with type 2 diabetes mellitus. *Diabetes Res. Clin. Pract.* 106 465–473. 10.1016/j.diabres.2014.09.039 25451914

[B35] Segura B. Baggio H. C. Solana E. Palacios E. M. Vendrell P. Bargallo N. (2013). Neuroanatomical correlates of olfactory loss in normal aged subjects. *Behav. Brain Res.* 246 148–153. 10.1016/j.bbr.2013.02.025 23458742

[B36] Shemesh D. Bokobza N. Rozenberg K. Rosenzweig T. Abookasis D. (2019). Decreased cerebral blood flow and hemodynamic parameters during acute hyperglycemia in mice model observed by dual-wavelength speckle imaging. *J. Biophotonics* 12:e201900002. 10.1002/jbio.201900002 30950209

[B37] Wang Y. Sun L. He G. Gang X. Zhao X. Wang G. (2021). Cerebral perfusion alterations in type 2 diabetes mellitus - a systematic review. *Front. Neuroendocrinol.* 62:100916. 10.1016/j.yfrne.2021.100916 33957174

[B38] Xia W. Rao H. Spaeth A. M. Huang R. Tian S. Cai R. (2015). Blood pressure is associated with cerebral blood flow alterations in patients with T2DM as revealed by perfusion functional MRI. *Medicine* 94:e2231. 10.1097/MD.0000000000002231 26632913 PMC4674216

[B39] Xu G. Rowley H. A. Wu G. Alsop D. C. Shankaranarayanan A. Dowling M. (2010). Reliability and precision of pseudo-continuous arterial spin labeling perfusion MRI on 3.0 T and comparison with 15O-water PET in elderly subjects at risk for Alzheimer’s disease. *NMR Biomed.* 23 286–293. 10.1002/nbm.1462 19953503 PMC2843795

[B40] Yan R. Tao S. Liu H. Chen Y. Shi J. Yang Y. (2019). Abnormal alterations of regional spontaneous neuronal activity in inferior frontal orbital gyrus and corresponding brain circuit alterations: a resting-state fMRI Study in Somatic Depression. *Front. Psychiatry* 10:267. 10.3389/fpsyt.2019.00267 31114515 PMC6503088

[B41] Yezhuvath U. S. Uh J. Cheng Y. Martin-Cook K. Weiner M. Diaz-Arrastia R. (2012). Forebrain-dominant deficit in cerebrovascular reactivity in Alzheimer’s disease. *Neurobiol. Aging* 33 75–82. 10.1016/j.neurobiolaging.2010.02.005 20359779 PMC2896562

[B42] Yoon S. Cho H. Kim J. Lee D. W. Kim G. H. Hong Y. S. (2017). Brain changes in overweight/obese and normal-weight adults with type 2 diabetes mellitus. *Diabetologia* 60 1207–1217. 10.1007/s00125-017-4266-7 28447116

[B43] Zald D. H. McHugo M. Ray K. L. Glahn D. C. Eickhoff S. B. Laird A. R. (2014). Meta-analytic connectivity modeling reveals differential functional connectivity of the medial and lateral orbitofrontal cortex. *Cereb. Cortex* 24 232–248. 10.1093/cercor/bhs308 23042731 PMC3862271

[B44] Zhang Z. Zhang B. Wang X. Zhang X. Yang Q. X. Qing Z. (2018). Altered odor-induced brain activity as an early manifestation of cognitive decline in patients with type 2 diabetes. *Diabetes* 67 994–1006. 10.2337/db17-1274 29500313

[B45] Zhang Z. Zhang B. Wang X. Zhang X. Yang Q. X. Qing Z. (2019). Olfactory dysfunction mediates adiposity in cognitive impairment of type 2 diabetes: insights from clinical and functional neuroimaging studies. *Diabetes Care* 42 1274–1283. 10.2337/dc18-2584 31221697

[B46] Zhou C. Li J. Dong M. Ping L. Lin H. Wang Y. (2021). Altered white matter microstructures in type 2 diabetes mellitus: a coordinate-based meta-analysis of diffusion tensor imaging studies. *Front. Endocrinol.* 12:658198. 10.3389/fendo.2021.658198 34012420 PMC8127836

[B47] Zhu J. Zhuo C. Qin W. Xu Y. Xu L. Liu X. (2015). Altered resting-state cerebral blood flow and its connectivity in schizophrenia. *J. Psychiatr. Res.* 63 28–35. 10.1016/j.jpsychires.2015.03.002 25812945

[B48] Zou Q. Wu C. W. Stein E. A. Zang Y. Yang Y. (2009). Static and dynamic characteristics of cerebral blood flow during the resting state. *Neuroimage* 48 515–524. 10.1016/j.neuroimage.2009.07.006 19607928 PMC2739419

