# Supplementary material for: Alterations of Cerebral Blood Flow and Its Connectivity in Olfactory-Related Brain Regions of Type 2 Diabetes Mellitus Patients
Source: Front Neurosci. 2022 Jul 11;16:904468. doi: 10.3389/fnins.2022.904468 (PMC9309479; doi:10.3389/fnins.2022.904468)
Supplement: Supplementary file 1 [file Table_1.DOCX]

**SUPPLEMENTARY TABLES**

**SUPPLEMENTARY TABLE 1 |** Correlation of the CBF changes in olfactory brain regions with clinical parameters and neuropsychological tests in patients with T2DM

| Index | Right OIFG | |  | Right Insula | |  | Left Olfactory cortex | |  | Right Olfactory cortex | |  |
| --- | --- | --- | --- | --- | --- | --- | --- | --- | --- | --- | --- | --- |
|  | *r* | *P* |  | *r* | *P* |  | *r* | *P* |  | *r* | *P* | |
| FPG | 0.167 | 0.180 |  | 0.139 | 0.267 |  | 0.035 | 0.780 |  | -0.035 | 0.777 | |
| HbA1c | 0.189 | 0.256 |  | 0.261 | 0.136 |  | 0.129 | 0.302 |  | 0.152 | 0.296 | |
| gluMean | 0.015 | 0.942 |  | -0.305 | 0.130 |  | -0.309 | 0.125 |  | -0.405 | 0.040^*^ | |
| gluMax | 0.012 | 0.955 |  | -0.445 | 0.026^*^ |  | -0.329 | 0.109 |  | -0.366 | 0.072 | |
| gluMin | 0.250 | 0.228 |  | 0.147 | 0.484 |  | 0.356 | 0.081 |  | 0.271 | 0.190 | |
| CV | -0.127 | 0.535 |  | 0.008 | 0.969 |  | -0.045 | 0.827 |  | 0.034 | 0.868 | |
| MAGE | -0.142 | 0.488 |  | -0.056 | 0.784 |  | -0.093 | 0.653 |  | -0.249 | 0.220 | |
| CSIT | -0.040 | 0.650 |  | -0.030 | 0.738 |  | 0.075 | 0.396 |  | 0.102 | 0.249 | |
| MoCA | -0.013 | 0.888 |  | -0.097 | 0.276 |  | -0.006 | 0.942 |  | 0.046 | 0.602 | |
| TMT-A | -0.052 | 0.561 |  | -0.045 | 0.614 |  | 0.013 | 0.888 |  | -0.050 | 0.577 | |
| DST-backward | 0.066 | 0.459 |  | 0.031 | 0.726 |  | 0.132 | 0.137 |  | 0.113 | 0.204 | |
| SDMT | -0.095 | 0.287 |  | -0.077 | 0.386 |  | -0.066 | 0.462 |  | -0.035 | 0.692 | |
| VFT-animal | -0.019 | 0.834 |  | 0.024 | 0.787 |  | -0.069 | 0.439 |  | -0.038 | 0.672 | |
| VFT-fruit | 0.020 | 0.821 |  | 0.010 | 0.913 |  | 0.069 | 0.441 |  | 0.099 | 0.265 | |
| SDS | 0.247 | 0.045^*^ |  | 0.149 | 0.233 |  | -0.128 | 0.305 |  | -0.145 | 0.244 | |

Note: CBF, cerebral blood flow; T2DM, type 2 diabetes mellitus; OIFG, orbital part of the inferior frontal gyrus; FPG, Fasting plasma glucose; HbA1c, Glycated hemoglobin; gluMean, Mean blood glucose; gluMax, Max blood glucose; gluMin, Minimum blood glucose; CV, Coefficient of variation; MAGE, Mean amplitude of glycemic excursions; CSIT, Chinese Smell Identification Test; MoCA, Montreal Cognitive Assessment; TMT-A, Trail Making Test-A; DST-backward, Digit Span Test-backward; SDMT, Symbol Digital Modalities Test; VFT-animal, Verbal Fluency Test-animal; VFT-fruit, Verbal Fluency Test-fruit; SDS, Self-Rating Depression Scale; * , *P* value < 0.05.

**SUPPLEMENTARY TABLE 2 |** Comparison of disease duration in subgroups of T2DM

| Index | Non-drug therapy | Antidiabetic drug therapy | *P* value |
| --- | --- | --- | --- |
| Duration of diabetes (months), median (25th, 75th percentile) | 12 (2, 27) | 48 (24, 108) | < 0.001 |

Note: T2DM, type 2 diabetes mellitus. Mann-Whitney U test for non-normal distribution data.
